# Acute bilateral vision and hearing loss following high-dose selenium ingestion: a case report

**DOI:** 10.3389/fphar.2026.1854419

**Published:** 2026-08-05

**Authors:** Xueping Liu, Huiting Liu, Yuquan Chen, Zhiqian Yang, Yifan Ye, Yongxiang Tang, Yingying Huang, Zhi Wang

**Affiliations:** Twelfth Guangzhou City People’s Hospital, Guangzhou, China

**Keywords:** blindness, case report, deafness, optic nerve injury, selenium toxicity

## Abstract

**Background:**

Selenium is an essential trace element widely used in health supplements. Reported cases of selenium poisoning have mainly involved hair loss, nail changes, and liver dysfunction, whereas toxic encephalopathy is uncommon. To date, no cases of selenium poisoning causing vision or hearing loss have been reported.

**Case Presentation:**

We report a 27-year-old woman employed in health supplement research and development who was occupationally exposed to multiple selenium compounds. During product testing in April 2025, she ingested selenium-containing samples. Based on the product formulation and the recorded amount sampled, the estimated oral selenium intake was 40.146 mg; however, this value represents an exposure estimate rather than a directly measured absorbed dose. She subsequently developed multisystem toxicity, including toxic encephalopathy, bilateral vision loss, hearing loss, liver dysfunction, alopecia, nail changes, and radial bone marrow edema. Initial symptoms were nausea and vomiting, followed by hearing loss, impaired consciousness, vision loss, and liver dysfunction. The first post-exposure blood selenium concentration was 13,131.8 ng/mL, and the urinary selenium concentration was 20,756.1 ng/mL.

**Intervention and Outcome:**

Treatment included sedation, diuretics, corticosteroids, hyperbaric oxygen therapy, organ protection, neurotrophic support, anti-infective therapy, and nutritional support. Consciousness recovered within 2 weeks, and liver function normalized. Vision transiently improved but later worsened. At 35-week follow-up, the right eye had no light perception, the left eye had visual acuity of 20 cm, and hearing loss had progressed to complete deafness.

**Conclusion:**

Extremely high-dose selenium ingestion can cause toxic encephalopathy and previously unreported persistent vision and hearing loss. Early supportive treatment and measures promoting selenium elimination may improve some clinical outcomes.

## 1 Introduction

Selenium is a nonmetallic chemical element belonging to Group 16 of the periodic table. As an essential element for maintaining the physiological health of livestock and humans, selenium (Se) participates in multiple vital biological functions, including antioxidant defense, thyroid hormone metabolism, and immune regulation (Rayman et al., 2008; Ellwanger et al., 2016; Holben and Smith, 1999; Pitts et al., 2014). Clinically, selenium supplements are used as adjunctive therapies for HIV patients (Stone et al., 2010), Crohn’s disease patients (Kuroki et al., 2003), cardiovascular disease patients (Benstoem et al., 2015), thyroid disease patients (Wu et al., 2015), and other conditions. Health Canada has approved 16 forms of selenium for use in licensed nutritional supplements, with commonly used forms including sodium selenite and yeast-derived selenium (LeBlanc and Mester, 2021; Ferrari et al., 2023). Consequently, selenium supplements are extensively used in both lifestyle wellness and clinical medicine. Personnel involved in the research, development, and production of selenium supplements face greater opportunities for inhalation and ingestion of selenium compounds compared to the general population. The patient described in this report was involved in selenium supplement development and ingested selenium-containing samples during product testing. According to the product formulation and the recorded amount sampled, the estimated oral selenium intake on that day was 40.146 mg. This value should be interpreted as an exposure estimate rather than a directly measured absorbed dose. She subsequently developed severe toxic encephalopathy accompanied by vision and hearing loss. Initial blood selenium levels measured 13,131.8 ng/mL (equivalent to 13,131.8 μg/L after conversion), with urinary selenium at 20,756.1 ng/mL. An epidemiological study in India reported serum selenium concentrations in the general population ranging from 111 to 400 μg/L (Chawla et al., 2020). A Spanish study similarly documented serum selenium levels between 74 and 213 μg/L and urinary selenium concentrations of 1.5–108 μg/L (Bocca et al., 2019). The selenium concentrations measured in both blood and urine of this patient were markedly higher than those reported in the above populations. Literature reports indicate that symptoms of selenium and its compound poisonings commonly manifest as hair loss, nail changes (Bawaskar and Bawaskar, 2025; Senthilkumaran et al., 2012; Civas and Akpınar, 2024), digestive system symptoms (MacFarquhar et al., 2010), and liver function impairment (Urbankova et al., 2021; Hadrup and Ravn-Haren, 2023; Yu et al., 2023). Toxic encephalopathy is relatively rare (Naderi et al., 2021; Lei et al., 2024), and high-concentration exposure can lead to rapid death (Spiller et al., 2020). This patient survived despite high-concentration exposure. Furthermore, no previous case reports have described selenium poisoning causing both hearing loss and vision loss, highlighting the uncertainty regarding the toxic mechanisms of high-concentration selenium poisoning. At the 35-week follow-up, the patient’s vision and hearing had not recovered.

## 2 Case description

### 2.1 Exposure process

The patient was a 27-year-old woman employed in the research and development department of a health supplement company in Guangzhou. She had no known previous ocular, auditory, neurological, hepatic, renal, or endocrine disease. She worked a single-shift schedule of 8 h per day and 5 days per week. In April 2025, while participating in the development of a new selenium-containing supplement, she sampled the product twice on April 29, at 9:00 AM and 1:00 PM, to evaluate its taste. The samples contained selenomethionine, L-selenomethylcysteine, and selenium-enriched yeast.

The estimated oral selenium intake was calculated as 40.146 mg based on the selenium concentration in the product formulation and the recorded amount sampled. This value was not obtained from direct measurement of the absorbed dose and should therefore be interpreted as an estimated exposure dose. Because detailed manufacturing and quality-control records were incomplete, the exact reason for the extraordinarily high selenium concentration in the sample could not be fully determined. The product was not a routine commercially marketed supplement but an experimental formulation being evaluated during product development.

### 2.2 Exposure assessment and exclusion of alternative causes

The exposure was considered occupational rather than environmental because the patient worked in the research and development department of a health supplement company and had routine workplace contact with selenium-containing raw materials, including selenomethionine, Se-methyl-L-selenocysteine, selenium-enriched yeast, and sodium selenite. A workplace investigation showed that Se-methyl-L-selenocysteine, with a reported purity of 97.6%, was one of the company’s raw and auxiliary materials, with an annual use of approximately 1.4 kg. According to the available history, no comparable selenium exposure was identified in the patient’s home environment, usual diet, or personal supplement use.

In April 2025, during the development of a new product, the available selenomethionine raw material was insufficient. A 120-mg raw material labeled as “selenium” was therefore used instead of selenomethionine in the experimental formulation. On April 29, after the sample had been prepared, the patient performed routine product taste testing and subsequently developed acute symptoms. Based on the product formulation and the recorded amount sampled, the estimated oral selenium intake was 40.146 mg. However, this value represents an estimated external exposure dose rather than a directly measured absorbed dose. During hospitalization, a comprehensive clinical evaluation was conducted, including neurological, audiological, and ophthalmological assessments, to characterize the patient’s nervous system injury, hearing impairment, and visual impairment and to exclude major alternative causes. The neurological evaluation included cerebrospinal fluid next-generation sequencing and brain magnetic resonance imaging. Audiological assessments included pure-tone audiometry, acoustic immittance testing, otoacoustic emission testing, stapedial reflex decay testing, auditory brainstem response testing, and auditory steady-state response testing. Ophthalmological assessments included flash visual evoked potentials, flash electroretinography, optical coherence tomography, and specialized optic disc examination. Overall, these findings did not support hearing or visual impairment attributable to pre-existing disease or previous medical history. Other potential causes of acute bilateral vision and hearing loss were also considered. The patient had no known previous ocular, auditory, neurological, hepatic, renal, or endocrine disease. There was no known history of chronic selenium supplementation, non-occupational heavy metal exposure, alcohol or illicit drug use, or long-term medication use. The available clinical evaluation did not support infectious encephalitis, cerebrovascular disease, traumatic injury, hereditary optic neuropathy, or drug-induced ototoxicity from other known agents. Taken together, the close temporal relationship between workplace taste testing of a selenium-containing sample, the markedly elevated blood and urinary selenium concentrations, and the subsequent multisystem injury supported acute occupational selenium intoxication as the most likely diagnosis.

### 2.3 Disease progression

**FIGURE 1:**
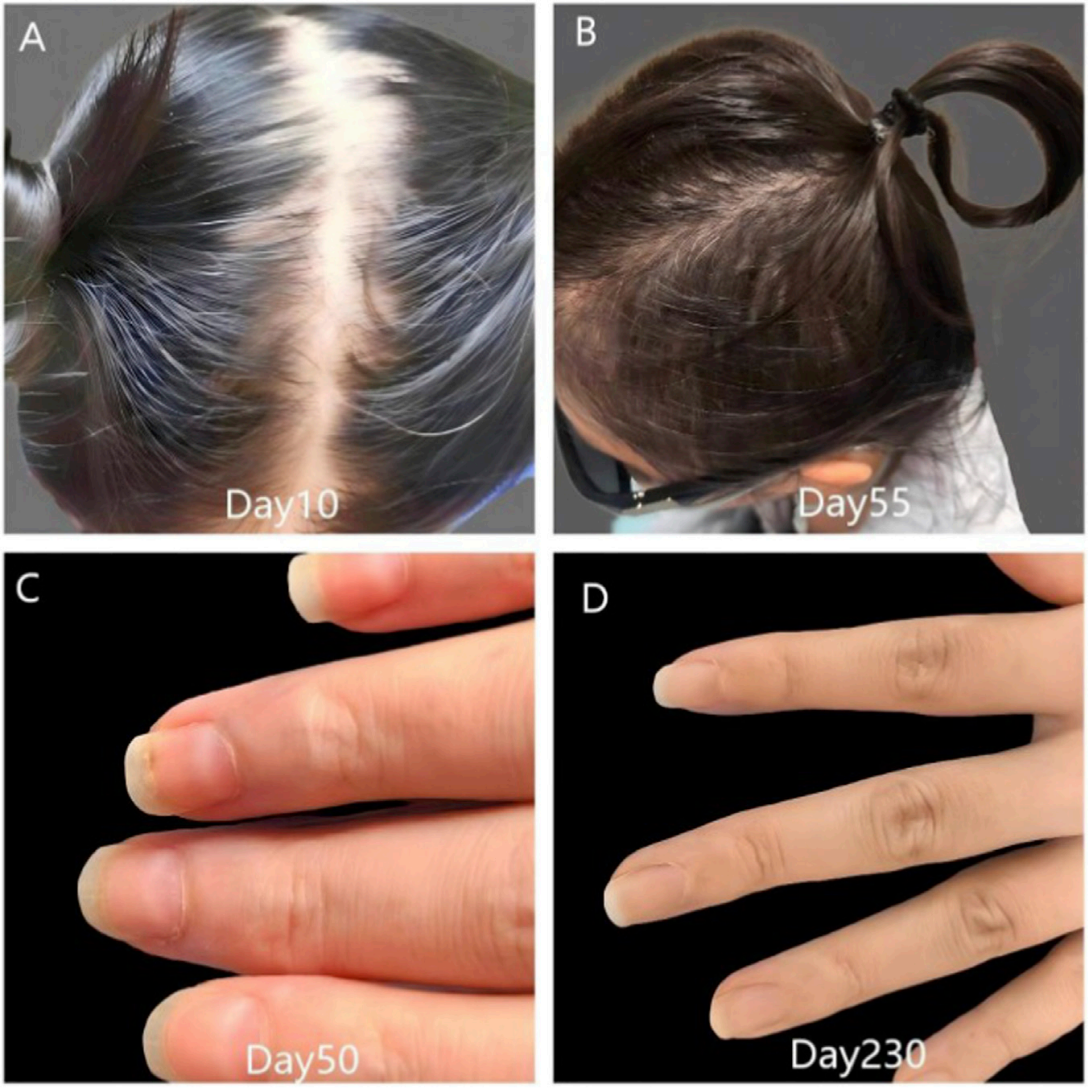
Dermatological manifestations after selenium intoxication. **(A)** Significant alopecia on May 9. **(B)** Decreased hair loss and new hair growth on May 21. **(C)** Mees’ lines on the fingernails on June 15. **(D)** Disappearance of Mees’ lines with new nail growth on December 15.

**FIGURE 2:**
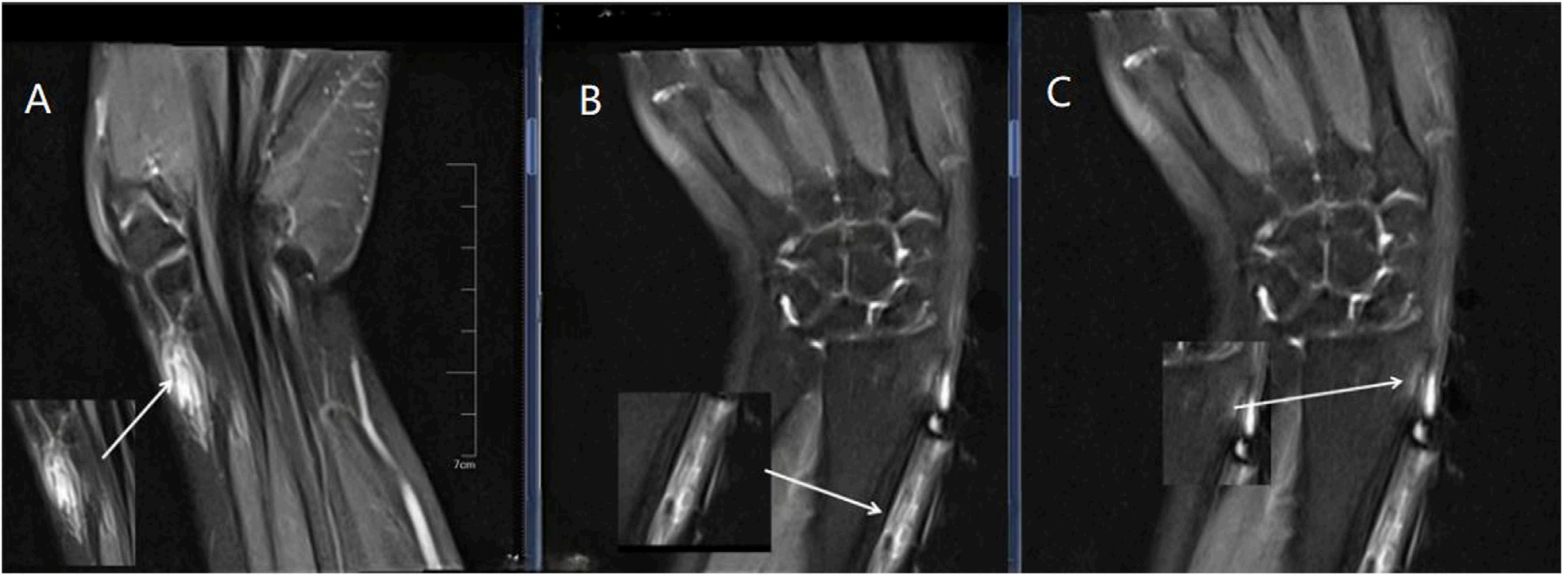
Wrist MRI findings. **(A)** Soft tissue edema on the radial side of the left wrist. **(B)** Soft tissue edema on the radial side of the right wrist. **(C)** Mild bone marrow edema in the distal right radius.

**FIGURE 3:**
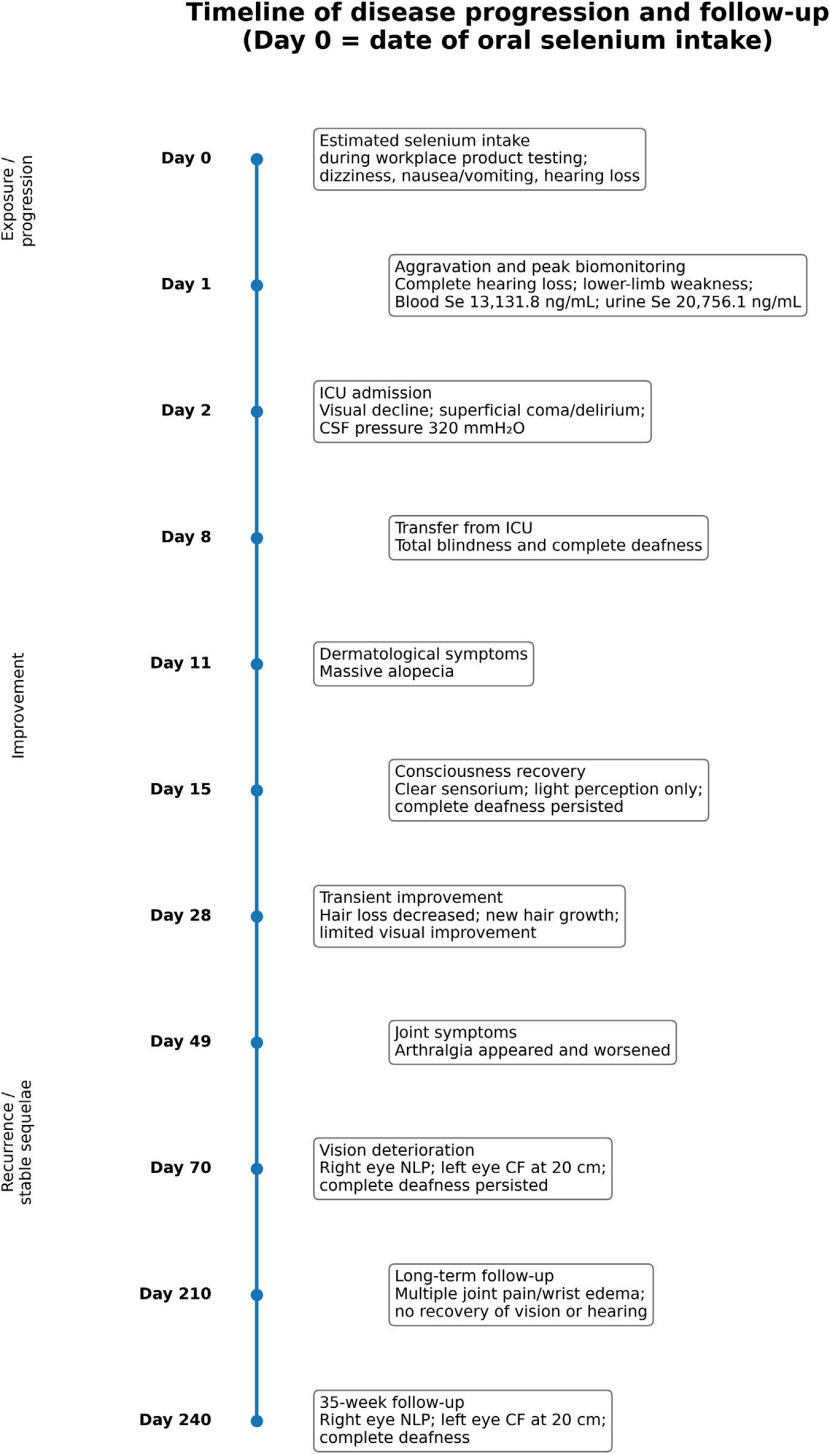
Timeline of the patient’s disease progression from exposure to treatment outcome. Day 0 represents the date of oral selenium intake.

The patient experienced nausea accompanied by dizziness at 2:00 PM on April 29. Around 9:00 PM, hearing loss developed, though loud verbal communication remained possible. Hearing deterioration progressively worsened, culminating in complete hearing loss by 8:00 AM on April 30. The patient remained alert and could communicate via written notes. Lower limb weakness developed, rendering walking impossible. Objective audiological assessment at a local hospital showed absent auditory brainstem response (ABR) waves I, III, and V bilaterally in response to click stimuli at the maximum output of 96 dB nHL, supporting severe bilateral auditory pathway dysfunction. At 14:00 on April 30, the patient developed impaired consciousness, presenting with a shallow coma and delirium. She was admitted to the intensive care unit (ICU) of Guangzhou Occupational Disease Prevention Hospital in the early hours of May 1 because of “nausea and vomiting for one and a half days, hearing loss for 29 h, and coma for 13 h” Admission examination: T (temperature): 36.8 °C, P (pulse): 93 bpm, R (respiratory rate): 21 bpm, BP (blood pressure): 98/73 mmHg. Patient in a state of shallow coma. Bilateral pupils: equal in size and round, diameter 6.0 mm, with sluggish pupillary light reflexes. Unable to cooperate with visual acuity testing. Rough hearing test showed no response. Neck was soft with no resistance, no jugular vein distension observed. Coarse breath sounds in both lungs, slight moist rales heard bilaterally, no dry rales heard. Regular heart rhythm, no extra heart sounds heard. Abdomen was soft and flat, no tenderness or rebound tenderness, no abdominal mass palpable, liver and spleen not palpable below the costal margin, bowel sounds normal. No edema in extremities. Muscle tone normal in limbs. Uncooperative during muscle strength testing. Physiological reflexes present. Pathological signs absent. Previous abnormal test results include: CK-MB 2.95 ng/mL, CK 1119 U/L, AST 37 U/L, ALT 66 U/L, Cr 63 μmol/L. Complete blood count: WBC 12.65 × 10^9/L, NEUT 85.4%, HB 127 g/L, PLT 200 × 10^9/L. Admission diagnosis: 1. Toxic encephalopathy 2. Hearing loss 3. Hepatic insufficiency 4. Abnormal cardiac enzymes. Upon admission, cerebrospinal fluid pressure measured 320 mmH_2_O. Treatment included dehydration therapy to reduce intracranial pressure, corticosteroids for anti-inflammation, neurotrophic agents, and dimercaptopropanesulfonic acid (DMPS) to chelate toxins. The patient’s mental status improved but remained delirious. Transferred to a general ward on May 6 for continued treatment, presenting with complete blindness and deafness at transfer. Massive hair loss began on May 9 (Figure 1). Wrist MRI later showed soft-tissue edema on the radial sides of both wrists and mild bone marrow edema in the distal right radius (Figure 2). The patient’s multisystem manifestations and their duration are summarized in Table 1, serial post-exposure changes in vision and hearing are summarized in Table 2, and the overall clinical course is shown in Figure 3.

**TABLE 1:** Clinical manifestations and duration of damage in each system.

System involved	Nervous system	Gastrointestinal system	Visual system	Auditory system	Bone and joint	Endocrine system	Others - hair	Others - nails
Clinical manifestations	Coma; delirium	Nausea, vomiting, hepatic dysfunction	Visual impairment	Hearing loss (deafness)	Arthralgia; edema	Decreased ACTH and cortisol levels	Alopecia	Mees’ lines
Onset time	Day 1	Day 0	Day 3	Day 0	Day 47	Day 90	Day 11	Day 47
Resolution time	Day 15	Day 8	Not recovered	Not recovered	Not recovered	Day 240	Day 28	Day 210

**TABLE 2:** Overview of post-exposure changes in vision and hearing.

Time (post-exposure)	Vision assessment	Hearing assessment
Day 1	Normal	Hearing loss
Day 2	Normal	Complete hearing loss; ABR report: no waves I, III, or V elicited by click stimuli (maximum output 96 dB nHL) bilaterally
Day 3	Visual acuity decreased	Total deafness
Day 8	Total blindness	Total deafness
Day 15	OU: light perception (LP), no light projection	Total deafness
Day 22	OS: CF at 20 cm; OD: HM at 10 cm	Total deafness
Day 28	OS: CF at 40 cm; OD: CF at 30 cm	Total deafness
Day 41	OS: CF at 40 cm; OD: CF at 40 cm	Total deafness
Day 56	OD: CF at 10 cm; OS: CF at 30 cm	Total deafness
Day 70	OD: NLP (no light perception); OS: CF at 20 cm	Total deafness
Day 210	OD: NLP; OS: CF at 20 cm	Total deafness
Day 240	OD: NLP; OS: CF at 20 cm	Total deafness

### 2.4 Critical laboratory monitoring indicators

**FIGURE 4:**
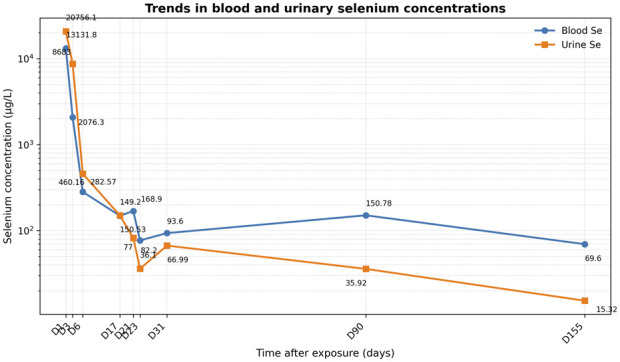
Trends in blood and urinary selenium concentrations after exposure.

Post-exposure monitoring revealed markedly elevated blood and urinary selenium concentrations in this patient. Blood and urinary selenium were measured using Inductively coupled plasma mass spectrometry (ICP-MS). The highest blood selenium concentration was 13,131.8 ng/mL on day 1 post-exposure, while the peak urinary selenium concentration reached 20,756.1 ng/mL on the same day. Both blood and urinary selenium concentrations peaked on the first day post-exposure and then showed a downward trend following treatment, with a more pronounced decline in urinary selenium. Blood selenium concentration decreased to the reference range by day 17 post-exposure (Figure 4). Monitoring also revealed abnormalities in transaminases (ALT/AST), plasma cortisol, plasma adrenocorticotropic hormone, white blood cell count, creatine kinase, and creatine kinase-MB. These parameters improved or returned to the reference range during follow-up (Table 3).

**TABLE 3:** Key laboratory indicators of the patient.

Item	Day 1	Day 2	Day 3	Day 9	Day 30	Day 50	Day 70	Day 90	Day 110	Day 130	Day 150	Day 240
CRE	62	50	43	34	39	37	43	42	39	43	45	75
CK	4,335	9,181	9,815	540	34	15	18	20	28	37	—	120
CK-MB	30.8	57.5	64.1	17.1	10.4	8.7	8.3	10.2	12.6	13.4	—	91.3
LDH	—	—	—	258	227	225	234	247	238	241	—	205
α-HBDH	—	—	—	204	191	177	181	194	188	198	—	155
AST	43	80	17	10	10	10	10	13	14	14	14	26
ALT	58	64	68	42	20	15	17	20	13	18	17	32
WBC	14.09	11.15	7.95	14.14	12.57	9.85	11.46	12.03	12.67	7.26	6.25	5.42
NEU	10.08	7.85	6.02	10.53	9.9	6.66	7.31	7.53	9.61	4.81	3.22	2.98
HGB	111	107	104	113	121	120	122	117	129	130	127	132
PLT	194	149	147	230	215	173	238	211	232	205	238	240
RENIN	—	—	—	—	—	—	—	28.69	17.67	7.43	8.73	50.76
ACTH	—	—	—	—	—	—	—	0.5	<0.220	<0.220	0.24	1.61
ALD	—	—	—	—	—	—	—	121.62	115.67	69.59	64.25	138.05
AII	—	—	—	—	—	—	—	85.29	81.53	82.85	74.82	112.04
ADRR	—	—	—	—	—	—	—	4.24	6.55	9.37	7.36	2.72
PC	—	—	—	—	—	—	—	116.98	3.47	<2.764	14.05	106.86

CRE, creatinine; CK, creatine kinase; CK-MB, creatine kinase-MB; LDH, lactate dehydrogenase; α-HBDH, α-hydroxybutyrate dehydrogenase; AST, aspartate aminotransferase; ALT, alanine aminotransferase; WBC, white blood cell count; NEU, neutrophil count; HGB, hemoglobin; PLT, platelet count; RENIN, renin concentration; ACTH, adrenocorticotropic hormone; ALD, aldosterone; AII, angiotensin II; ARR, aldosterone-renin ratio; PC, plasma cortisol.

## 3 Discussion

**FIGURE 5:**
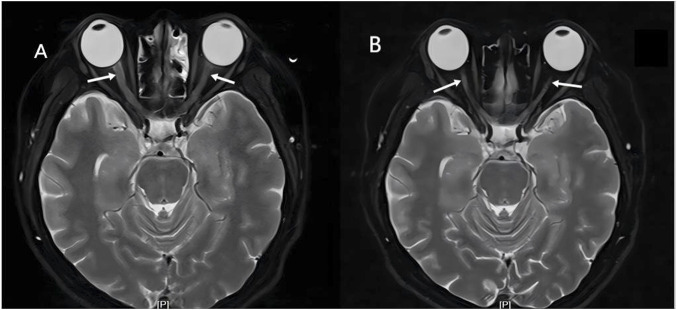
Day 9 post-exposure optic nerve MRI: bilateral optic nerve swelling with patchy T2WI hyperintense signals and marked contrast enhancement on contrast-enhanced imaging **(A)**. Day 154 post-exposure optic nerve MRI: optic nerve without swelling, slightly thinned, showing patchy T2WI mildly hyperintense signals; moderate contrast enhancement with reduced intensity on contrast-enhanced imaging **(B)**.

Selenium, as an essential trace element for the human body, is widely used in nutritional supplements in the form of selenium compounds. Although it is crucial as a trace essential nutrient for many organisms, its safety margin is extremely narrow—exceeding threshold concentrations renders selenium highly toxic (Mézes and Balogh, 2009). Symptoms of selenium poisoning include congenital defects, reproductive system damage, liver failure, skin lesions, respiratory failure, gastrointestinal discomfort, hair loss and nail changes, and fatigue. These phenomena have been documented in various organisms, including humans (Fordyce and Selinus, 2013; Spallholz, 1994; Vinceti et al., 2001). Previously, only a limited number of case reports documented toxic encephalopathy resulting from selenium and its compound poisoning. No cases of selenium poisoning leading to hearing or vision loss have been reported. According to the 2023 Edition of Dietary Reference Intakes for Chinese Residents, Part 3, the Estimated Average Requirement (EAR) for selenium in Chinese women aged 18 years and older is 50 μg/day, the Recommended Nutrient Intake (RNI) is 60 μg/day, and the Tolerable Upper Intake Level (UL) is 400 μg/day (Chinese Nutrition Society, 2023). Hadrup et al. confirmed that the lower threshold for selenium toxicity dose descriptors could be as low as 2–3 µg selenium/kg body weight/day (Hadrup and Ravn-Haren, 2023). The patient weighed 48 kg, and the estimated oral selenium exposure dose based on product formulation and recorded sampling amount was 40.146 mg, approximately 100 times the UL and 278 times the lower threshold for selenium toxicity. However, this value should not be interpreted as the directly measured absorbed dose or total body burden. The subsequent blood and urinary selenium concentrations were extremely high, and the discrepancy between the estimated intake and biological monitoring results indicates uncertainty in exposure reconstruction. Possible explanations include incomplete information on the exact sampled amount, non-uniform selenium distribution in the experimental formulation, additional inadvertent intake during product tasting, or differences between estimated external dose and internal selenium kinetics. Subsequently, multiple organ damage emerged, including toxic encephalopathy, vision loss, hearing loss, liver dysfunction, hair loss, nail changes, decreased hormone levels, and radial bone marrow edema. Long-term excessive selenium intake in humans has been proven to cause various health issues. The patient’s symptoms—toxic encephalopathy, liver dysfunction, hair loss, nail changes, decreased hormone levels, and radial bone marrow edema—are consistent with previously documented cases. For instance, multiple studies indicate that excessive dietary selenium exposure disrupts thyroid hormone system function while affecting growth hormone and sex steroid hormone secretion and metabolism (Vinceti et al., 2001; Brätter and Negretti de Brätter, 1996; Thor et al., 1989; Winther et al., 2015). No human case reports of vision or hearing loss due to selenium poisoning have been previously documented. Only in 2016 did Raine et al. observe visual impairment symptoms in zebrafish chronically exposed to selenomethionine, manifested as reduced escape responses and abnormal visual behavioral responses in offspring during phototaxis, visual tracking, and visual tracking tests (Raine et al., 2016). In this case, the patient rapidly developed hearing and vision impairment early in the disease course. Complete hearing loss occurred on day 1 post-exposure, and complete vision loss was confirmed on day 8 post-exposure. Vision loss may have occurred earlier, as the patient was comatose upon admission to our ICU and unable to cooperate with visual examinations. Optic nerve MRI performed on day 9 post-exposure revealed optic neuritis (Figure 5A). Despite treatment, the patient’s vision and hearing did not recover. Follow-up optic nerve MRI on day 154 post-exposure showed mild bilateral optic nerve atrophy with reduced contrast enhancement compared with the earlier scan (Figure 5B). This case demonstrates that selenium poisoning can cause irreversible optic nerve atrophy leading to vision loss. Although no imaging abnormalities of the auditory nerve were detected during the course of the disease, audiological test results suggest that the hearing loss is due to supranuclear hearing impairment. The site of injury may be located in the mid-auditory pathway above the brainstem auditory nuclei, such as the brainstem, pons, or midbrain. The specific mechanism of injury requires further investigation. Therefore, the association between selenium intoxication and persistent visual and auditory impairment in this case should be interpreted as a clinically plausible temporal association supported by biological monitoring, rather than definitive mechanistic proof. The highest blood selenium concentration measured in this patient was 13,131.8 ng/mL on day 1 post-exposure, while the peak urinary selenium concentration reached 20,756.1 ng/mL on the same day. A comparable level was reported in a 2020 U.S. case of fatal occupational selenium poisoning due to inhalation of L-selenomethionine, in which the patient had a blood selenium concentration of 11,000 μg/L and a urinary selenium concentration of 25,000 μg/L and died 5 h after exposure. The different outcome in the present case may be related to differences in exposure route, selenium compound, absorbed dose, clinical course, and treatment timing. The patient in this case received supportive therapy, corticosteroids, diuretics, and measures intended to promote selenium elimination. However, because this is a single case report, a causal relationship between specific treatments and clinical improvement cannot be established.

This study has several limitations that must be acknowledged. First, the ingested selenium dose was reconstructed from the product formulation and the recorded amount sampled; it was not a directly measured absorbed dose. Therefore, the reported value of 40.146 mg should be interpreted as an estimated exposure dose. Second, the workplace was not classified as a toxic chemical production site, and the patient had not undergone routine occupational health examinations before the event. Consequently, baseline blood and urinary selenium concentrations were unavailable. Third, although the available history supported occupational exposure rather than environmental exposure, complete environmental selenium measurements from the home were not available. Fourth, objective clinical data were limited by the patient’s early coma and severe sensory impairment. ABR testing and optic nerve MRI provided important objective evidence, although comprehensive neurological, audiological, and ophthalmic examinations were performed, the early assessment was partly limited by the patient’s impaired consciousness and severe sensory dysfunction. Therefore, some functional evaluations depended on objective electrophysiological and imaging findings rather than subjective patient responses. Finally, as a single case report, this study cannot establish causality or determine the precise mechanisms by which selenium intoxication may induce auditory and optic nerve injury. Further clinical and experimental studies are required to clarify these mechanisms.

## Data Availability

All data generated or analyzed during this study are included in this article. Additional data supporting the findings of this study are available from the corresponding author upon reasonable request.
